# Safety and tolerability of andecaliximab as monotherapy and in combination with an anti-PD-1 antibody in Japanese patients with gastric or gastroesophageal junction adenocarcinoma: a phase 1b study

**DOI:** 10.1136/jitc-2021-003518

**Published:** 2022-01-06

**Authors:** Akie Kimura Yoshikawa, Kensei Yamaguchi, Kei Muro, Atsuo Takashima, Takashi Ichimura, Daisuke Sakai, Shigenori Kadowaki, Keisho Chin, Toshihiro Kudo, Seiichiro Mitani, Shigehisa Kitano, Dung Thai, Marianna Zavodovskaya, JieJane Liu, Narikazu Boku, Taroh Satoh

**Affiliations:** 1 Department of Gastroenterology, Osaka City University Graduate School of Medicine, Osaka, Japan; 2 Department of Gastroenterology, Japanese Foundation for Cancer Research, Tokyo, Japan; 3 Department of Clinical Oncology, Aichi Cancer Center Hospital, Nagoya, Japan; 4 Division of Gastrointestinal Medical Oncology, National Cancer Center Hospital, Tokyo, Japan; 5 Department of Gastrointestinal Oncology, The Cancer Institute Hospital of Japanese Foundation for Cancer Research, Tokyo, Japan; 6 Department of Frontier Science for Cancer and Chemotherapy, Osaka University Graduate School of Medicine, Osaka, Japan; 7 Department of Gastroenterological Chemotherapy, Cancer Institute Hospital of Japanese Foundation for Cancer Research, Tokyo, Japan; 8 Department of Medical Oncology, Osaka International Cancer Institute, Osaka, Japan; 9 Department of Medical Oncology, Kindai University, Higashiosaka, Japan; 10 Department of Experimental Therapeutics, National Cancer Center Hospital, Goyang, Republic of Korea; 11 Gilead Sciences Inc, Foster City, California, USA

**Keywords:** gastrointestinal neoplasms, drug therapy, combination

## Abstract

**Background:**

Matrix metalloproteinase 9 (MMP9) is implicated in protumorigenic processes. Targeting either stromal or epithelial MMP9 reduces the incidence of metastasis. Andecaliximab is a monoclonal antibody that targets MMP9 with high affinity and selectivity. However, no study has examined whether the inhibition of T-cell programmed death 1 (PD-1) in the presence of andecaliximab increases activated lymphocyte infiltration into the tumor, thereby increasing antitumor activity more than that in anti-PD-1 monotherapy. In this study, we assessed the safety, pharmacokinetics (PK), exploratory biomarkers, and preliminary efficacy of andecaliximab as monotherapy and in combination with nivolumab in Japanese patients with advanced or recurrent gastric or gastroesophageal junction (GEJ) adenocarcinoma.

**Methods:**

This phase 1b study comprised four cohorts enrolling Japanese patients with gastric or GEJ adenocarcinoma. This paper concerns cohorts 1 and 4; cohorts 2 and 3 will be reported subsequently. Cohort 1 enrolled patients with human epidermal growth factor receptor 2 (HER2)-negative tumors (n=8) who received andecaliximab monotherapy (800 mg by intravenous infusion every 2 weeks (Q2W)), and cohort 4 enrolled patients irrespective of their HER2 status (n=10) who received 800 mg of andecaliximab in combination with nivolumab Q2W. Safety, dose-limiting toxicities (DLTs), PK, pharmacodynamics, and biomarkers were assessed in both cohorts.

**Results:**

PK of andecaliximab in Japanese patients with gastric or GEJ adenocarcinoma was similar to that reported in non-Japanese patients with advanced solid tumors. Andecaliximab monotherapy and in combination with nivolumab demonstrated no DLTs in cohort 1 and 4, respectively. Toxicities were manageable and well tolerated in both cohorts. The median progression-free survival was 1.4 months (90% CI, 0.5 to 5.4) and 4.6 months (90% CI, 0.9 to not reached) in cohorts 1 and 4, respectively. The objective response rate was 50% (90% CI, 22% to 78%) in cohort 4, and in some patients, the combination therapy was effective regardless of the biomarker status.

**Conclusions:**

The andecaliximab–nivolumab combination demonstrated a manageable safety profile and promising clinical activity in patients with advanced gastric adenocarcinoma.

NCT02862535.

## Background

Matrix metalloproteinases (MMPs) are members of a family of at least 23 Zn^2+^-dependent proteases that are involved in the degradation and remodeling of the extracellular matrix and basement membrane, as well as other growth factors, cytokines, and chemokines, during normal and pathologic biological processes.^1^ MMP9 is an extracellular enzyme involved in matrix remodeling during tumor growth and metastasis, and it is a poor prognostic factor for gastric cancer. MMP9 is an inducible MMP expressed heterogeneously by tumor epithelia, macrophages, neutrophils, other inflammatory cells, fibroblastic stroma, and tumor-associated endothelial cells. MMP9 activation can release cytokines, growth factors, and bioactive protein fragments that modulate inflammation, neovascularization, and matrix remodeling.^2–4^

The activity of MMP9 in the tumor microenvironment has been investigated in several cancer models.^5 6^ Preclinical studies have suggested that MMP9 inhibition can reverse immune suppression, promote T-cell infiltration, and potentiate checkpoint blockade.^7 8^ In patients with gastric cancer, elevated expression levels of MMP9 protein and mRNA are associated with reduced overall survival (OS).^9–11^ Andecaliximab is a recombinant chimeric IgG4 monoclonal antibody that demonstrates high affinity and selectivity for MMP9. The safety and tolerability of andecaliximab in patients with advanced solid tumors were validated in a phase 1 study (ClinicalTrials.gov identifier: NCT01803282). Thereafter, we initiated a study to evaluate the efficacy of andecaliximab combination chemotherapy and andecaliximab monotherapy in patients with gastric cancer (online supplemental figure 1).^12^

10.1136/jitc-2021-003518.supp3 Supplementary data

Nivolumab, a human monoclonal antibody directed against programmed death-1 (PD-1), blocks the interaction between PD-1 and programmed death ligand-1 (PD-L1) and is effective in patients with advanced gastric cancer.^13^ The inhibition of T-cell PD-1 in the presence of andecaliximab may increase the infiltration of activated lymphocytes into the tumor, thereby increasing antitumor activity more than anti-PD-1 monotherapy. Based on this idea, we hypothesized that a significant effect could be expected from the combination of anti-MMP9 with anti-PD-1 therapy. Thus, we also initiated this phase 1b study wherein we combined andecaliximab with an immune checkpoint inhibitor, anticipating advantages over chemotherapy in terms of clinical response and adverse event (AE) occurrence. In this study, we aimed to assess, for the first time, the safety, pharmacokinetics (PK), exploratory biomarkers, and preliminary efficacy of andecaliximab alone and in combination with nivolumab in Japanese patients with advanced or recurrent gastric or gastroesophageal junction (GEJ) adenocarcinoma.

## Methods

### Study design

This phase 1b, open-label, multicenter study comprised four cohorts: one monotherapy cohort and three combination therapy cohorts. This report concerns cohort 1 (andecaliximab monotherapy) and cohort 4 (combination therapy of andecaliximab and nivolumab). The results from cohort 2 (combination therapy of andecaliximab and S-1 plus cisplatin) and cohort 3 (combination therapy of andecaliximab and S-1 plus oxaliplatin) will be subsequently reported.

### Dosing

#### Cohort 1

Initially, six patients were scheduled to receive andecaliximab 800 mg via intravenous infusion over approximately 30 (±5) min every 2 weeks (Q2W). If two or more patients within the cohort experienced dose-limiting toxicities (DLTs) during the first 28 days of dosing, up to six additional patients were scheduled to receive andecaliximab at a reduced dose of 600 mg Q2W. If two additional DLTs occurred at a dose of 600 mg, andecaliximab was considered unsafe and was discontinued.

#### Cohort 4

Up to 10 patients were scheduled to receive andecaliximab 800 mg via intravenous infusion over approximately 30 (±5) min Q2W and nivolumab 3 mg/kg Q2W via intravenous infusion over 60 (±5) min after completing andecaliximab administration. The dose of nivolumab was adjusted if the patient’s weight changed by more than 10% of the baseline dosing weight.

### Patient eligibility

The inclusion criteria were as follows: age ≥20 years; Eastern Cooperative Oncology Group (ECOG) performance status ≤1; adequate baseline organ function (within 28 days before day 1 of antitumor treatment); born in Japan and not having lived outside of Japan for >1 year in the 5 years before day 1; traceable maternal and paternal ancestry of parents and grandparents as ethnically Japanese; and histologically confirmed unresectable advanced gastric adenocarcinoma (including GEJ adenocarcinoma) or recurrent gastric adenocarcinoma. Only patients with human epidermal growth factor receptor 2 (HER2)-negative tumors were included in cohort 1. For cohort 4, patient selection was not restricted by HER2 status. For cohort 1 patients, prior antitumor therapy or cytotoxic chemotherapy was acceptable. The prior antitumor therapy is shown in online supplemental table 1.

10.1136/jitc-2021-003518.supp1 Supplementary data

The exclusion criterion was radiotherapy within 28 days before day 1. For cohort 1 patients, we considered antitumor therapy within 28 days or five times half-lives of antitumor agents in immediately prior chemotherapy (exception: 6 weeks for nitrosoureas, mitomycin C, or molecular agents with a half-life >10 days), whichever was shorter, before day 1. In addition to the criterion above, patients who met any of the following exclusion criteria were not enrolled in cohort 4: received only neoadjuvant or adjuvant therapy for gastric adenocarcinoma; chronic daily treatment with oral corticosteroids (dose >10 mg/day prednisone equivalent) or other immunosuppressive medications within 14 days before day 1; antitumor therapy (chemotherapy, antibody therapy, or molecular targeted therapy) within 28 days or five times of half-lives of antitumor agents in immediately prior chemotherapy (6 weeks for nitrosoureas, mitomycin C, or molecular agents with a half-life >10 days), whichever was shorter, before day 1; prior treatment with anti-cytotoxic T-lymphocyte-associated protein 4 agents (eg, ipilimumab), anti-PD-1 or anti-PD-L1 agents (eg, pembrolizumab, nivolumab, or atezolizumab), anti-programmed cell death ligand 2 agents, anti-MMP agents, or other immunomodulatory therapies; prior therapy with antitumor vaccines or other immunomodulatory antitumor agents; current or history of pneumonitis or interstitial lung disease; active known or suspected autoimmune disease; and a history of bone marrow, stem cell, or allogenic organ transplantation.

### Study treatment

For cohorts 1 and 4, each antitumor treatment cycle was repeated every 28 days and continued in the absence of disease progression, unacceptable toxicity, withdrawal of consent, or other reasons.

Andecaliximab was administered at 800 mg via intravenous infusion over approximately 30 min Q2W. Nivolumab was administered at 3 mg/kg via intravenous infusion over approximately 60 min Q2W after completing andecaliximab administration.

### Endpoints

The primary objective of this study was to characterize the safety and tolerability of andecaliximab as monotherapy and in combination with nivolumab in Japanese patients with unresectable advanced or recurrent gastric or GEJ adenocarcinoma. The secondary objective of this study was to characterize the PK of andecaliximab. The exploratory objectives were to evaluate andecaliximab pharmacodynamic (PD) biomarkers and other markers of antitumor activities in the blood, explore biomarkers in tumor tissue, and evaluate the patient therapeutic response to andecaliximab as monotherapy and in combination with nivolumab.

### Efficacy

The exploratory efficacy endpoints were defined as follows. The objective response rate (ORR) was defined as the proportion of patients with complete response (CR) or partial response (PR) based on the RECIST V.1.1 as a best overall response during andecaliximab therapy. The disease control rate was defined as the proportion of patients with a best overall response after andecaliximab therapy of CR, PR, stable disease, or non-CR/non-PD based on RECIST V.1.1. Progression-free survival (PFS) was defined as the time from the administration of the first andecaliximab dose to the first documented occurrence of either definitive disease progression based on the RECIST V.1.1 or death from any cause. OS was defined as the time from the administration of the first andecaliximab dose to death from any cause.

### Pharmacokinetics

Plasma samples were collected and analyzed to assess andecaliximab concentrations and anti-andecaliximab antibody levels. For cohort 1 patients, to assess andecaliximab PK, we collected plasma samples before administering andecaliximab and 30 (±15) min after the end of infusion on day 1 of cycles 2, 3, 5, and 7 and every three cycles thereafter, as well as anytime at the end of treatment (EOT) (if not conducted in the last 2 weeks) and end of study (EOS) visits. Additionally, plasma samples for andecaliximab PK were collected at the following time points in cycle 1 only: 30 (±15) min after the end of infusion on day 1; anytime on days 2, 4, and 8; prior to andecaliximab administration; and 30 (±15) min after the end of infusion on day 15. For cohort 4 patients, plasma samples for andecaliximab PK in cycle 1 were collected 30 (±15) min after completing andecaliximab infusion on day 1, anytime on day 8, prior to andecaliximab administration, and 30 (±15) min after the end of infusion on day 15. Additionally, plasma samples for andecaliximab PK were collected before andecaliximab administration and 30 (±15) min after the end of infusion on days 1 and 15 of cycles 2, 3, 5, and 7 and every three cycles thereafter, as well as anytime at the EOT (if not conducted in the last 2 weeks) and EOS visits.

For all cohorts, blood samples for anti-andecaliximab antibody analysis were collected before dosing on day 1 of cycles 1, 2, 3, 5, and 7 and every three cycles thereafter, at the EOT visit (if not conducted in the last 2 weeks), at the EOS visit, and at the 30-day safety follow-up visit. For cohort 4 patients, blood samples for anti-andecaliximab antibodies were also collected at the 5-month safety follow-up visit.

### Safety assessments

Safety was evaluated through clinical assessments, laboratory analyses, 12-lead ECG analyses, and assessments of the incidences of AEs. Safety assessments were performed before each andecaliximab infusion according to the Common Terminology Criteria for Adverse Events V.4.03.

### Biomarkers

Free MMP9 (not bound to andecaliximab) and total MMP9 (bound and free) were measured in platelet-poor plasma samples using custom, quantitative sandwich ELISAs pre-dose at cycle 1 day 1 (C1D1) and on-treatment, at trough, at C1D15 (week 2), C2D15 (week 6). Tumor biopsies were collected pre-dose before C1D1 and at week 6. Archival tissue samples were analyzed when baseline biopsy samples lacked tumor tissue. MMP9 coverage and total and andecaliximab-free MMP9 levels were determined with a custom ELISA using platelet-poor plasma. PD-L1 (28-8; DAKO) immunohistochemistry (IHC), mismatch repair (MMR) IHC, and Epstein–Barr virus (EBV) (EBER in situ hybridization) testing were performed at LabCorp (Covance). MMR status was determined through PMS2 and MSH6 IHC, and MMR deficiency was classified as the loss of one or more MMR proteins.

### Sample size considerations

The sample sizes of cohorts 1 and 4 were defined based on the following considerations: a sample size of six patients in cohort 1 provided a relatively high probability (>65%) to observe two or more patients with DLTs when the true underlying DLT probability is >33.3% at the initial dose level of 800 mg andecaliximab Q2W. Thus, up to six patients were enrolled in cohort 1 to be treated with this dose, and based on safety assessments, up to six more patients were to be enrolled in cohort 1 to receive 600 mg andecaliximab Q2W prior to proceeding with the combination therapy cohorts, including cohort 4. Based on the dose level defined by the responses in cohort 1, up to 10 patients were supposed to be enrolled in cohort 4.

The sample size of 10 in combination cohort was considered acceptable to evaluate safety (online supplemental table addition-1) and was able to align with the practical availability of patients at the time frame when the study was conducted. Besides, the sample size of 10 can provide reasonable CI of observed ORR, assuming the true ORR is 12% as reported for nivolumab monotherapy in Kang *et al*^13^ (online supplemental table addition-2).

## Results

### Patient characteristics

The first participant was screened on September 20, 2016. As of December 4, 2018, 8 and 10 patients were dosed in cohorts 1 and 4, respectively. The median patient ages were 67 years and 62 years in cohorts 1 and 4, respectively. A total of 14 patients (77.8%) were male. Baseline patient characteristics were generally similar between the cohorts (table 1). All patients received prior chemotherapy before dosing (median: 2 regimens, range: 1–4 regimens; see online supplemental table 1).

**Table 1:** Baseline patient characteristics

	Cohort 1 (n=8)	Cohort 4 (n=10)	Total (n=18)
Age, median years (range)	67 (27–68)	62 (38–77)	66 (27–77)
Male, n (%)	5 (62.5)	9 (90.0)	14 (77.8)
Screening ECOG PS, n (%)			
0	4 (50.0)	5 (50.0)	9 (50.0)
1	4 (50.0)	5 (50.0)	9 (50.0)
Primary tumor site, n (%)			
Gastric	7 (87.5)	10 (100.0)	17 (94.4)
Proximal	2 (25.0)	2 (20.0)	4 (22.2)
Distal	4 (50.0)	5 (50.0)	9 (50.0)
Other	1 (12.5)	3 (30.0)	4 (22.2)
GEJ	1 (12.5)	0	1 (5.6)
Differentiation, n (%)			
Well differentiated	1 (12.5)	1 (10.0)	2 (11.1)
Moderately differentiated	1 (12.5)	3 (30.0)	4 (22.2)
Poorly differentiated	4 (50.0)	6 (60.0)	10 (55.6)
Unknown	2 (25.0)	0	2 (11.1)
Number of prior regimens			
1	0	2 (20.0)	2 (11.1)
2	4 (50.0)	4 (40.0)	8 (44.4)
3	2 (25.0)	3 (30.0)	5 (27.8)
4	2 (25.0)	1 (10.0)	3 (16.7)

The safety analysis set includes patients who received at least one dose of andecaliximab.

ECOG status: 0=Fully active, able to perform all pre-disease activities without restriction; 1=Restricted in physically strenuous activity but ambulatory and able to perform light or sedentary work; and 2=Ambulatory and capable of self-care but unable to perform working activities (up and about more than 50% of waking hours)

ECOG PS, Eastern Cooperative Oncology Group performance status; GEJ, gastroesophageal junction.

### Pharmacokinetics

Table 2 shows a summary of PK parameters of andecaliximab for cohort 1. The median time to maximum concentration was 0.43 hour after the start of infusion. The median half-life was 7.54 days following the administration of an 800 mg dose.

**Table 2:** Summary of andecaliximab PK parameters

PK parameter, mean (%CV)	Cohort 1 (n=8)
C_max_ (µg/mL)	264 (21.4)
AUC_last_ (day×µg/mL)	1406 (25.4)
AUC_inf_ (day×µg/mL)	2227 (20.2)
t_1/2_ (day)*	7.54 (6.79, 9.60)
CL (mL/day)	370.9 (18.8)
V (mL)	4375 (22.0)

*Median (IQR).

AUC_inf_, area under the concentration vs time curve extrapolated to infinite time; AUC_last_, area under the concentration vs time curve to the last measurable concentration; CL, clearance; C_max_, the maximum observed drug concentration; PK, pharmacokinetics; t_1/2_, half time; V, distribution volume.

**Figure 1:**
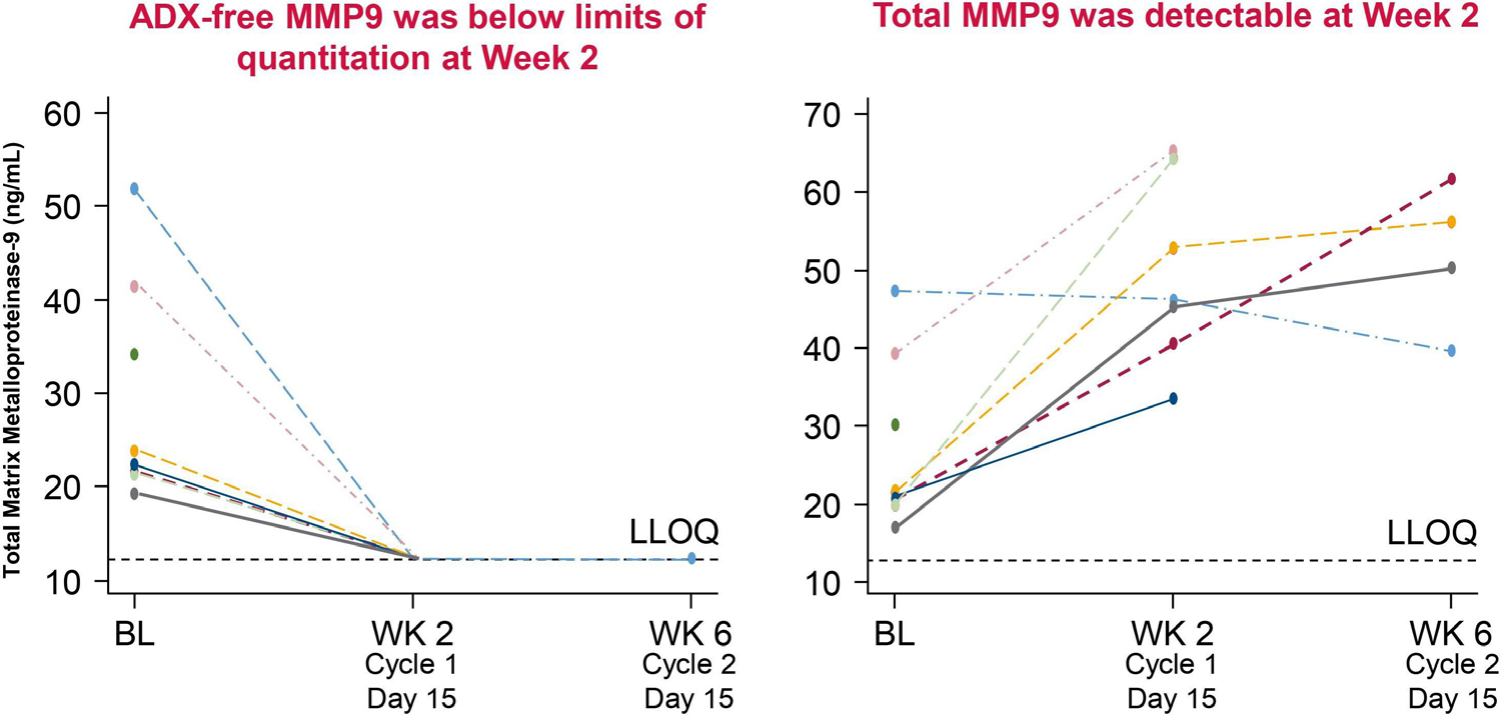
Pharmacodynamic evaluation of andecaliximab. andecaliximab-free matrix metalloproteinase 9 (ADX-free MMP9) was undetectable on day 15 of cycle 1; therefore, circulating MMP9 was bound to andecaliximab in week 2 after dosing in cohort 1 patients. Circulating free MMP9 levels were below the limits of quantitation on-treatment at trough, indicating that all MMP-9 was bound to andecaliximab for the duration of the Q2W dosing interval. The half-life of andecaliximab is 1 week; therefore, it can inhibit tumor growth as an anti-MMP9 agent. ADX, andecaliximab; MMP9, matrix metalloproteinase 9.

Total MMP9 and free MMP9 were measured in samples from cohort 1 undergoing monotherapy. At baseline, both total and free MMP9 were detectable. At trough, no free MMP9 was detectable, while total MMP9 was detectable at all time points and in all treated subjects, indicating that all circulating MMP9 was bound to andecaliximab on-treatment for the duration of the Q2W dosing interval (figure 1).

### Safety

Andecaliximab monotherapy and in combination with nivolumab demonstrated no DLTs in cohort 1 and 4, respectively. Four patients (40%) underwent ongoing andecaliximab treatment in cohort 4 on the last observation date of this report (December 4, 2018), but no patient in cohort 1 had ongoing andecaliximab treatment. The median total number of andecaliximab doses received per patient was 4. The median duration of exposure to andecaliximab was 7 weeks (range, 0–49 weeks) for all patients (n=18). Treatment-emergent AEs (TEAEs) occurring in all patients are shown in table 3. Fifty per cent (9/18) of patients experienced grade ≥3 TEAEs: 4/8 in cohort 1 and 5/10 in cohort 4.

**Table 3:** TEAEs of any grade observed in all patients

Event, n (%)	Cohort 1 (n=8)		Cohort 4 (n=10)		Total (n=18)		
	Grade 1–2	Grade 3–4	Grade 1–2	Grade 3–4	Grade 1–2	Grade 3–4	Any grade
Any TEAE	4 (50.0)	4 (50.0)	5 (50.0)	5 (50.0)	9 (50.9)	9 (50.0)	18 (100.0)
Nausea	4 (50.0)	0	2 (20.0)	0	6 (33.3)	0	6 (33.3)
Decreased appetite	3 (37.5)	1 (12.5)	1 (10.0)	1 (10.0)	4 (22.2)	2 (11.1)	6 (33.3)
Fatigue	2 (25.0)	1 (12.5)	2 (20.0)	1 (10.0)	4 (22.2)	2 (11.1)	6 (33.3)
Anemia	0	2 (25.0)	0	2 (20.0)	0	4 (22.2)	4 (22.2)
Fever	2 (25.0)	0	2 (20.0)	0	4 (22.2)	0	4 (22.2)
Malaise	3 (37.5)	0	1 (10.0)	0	4 (22.2)	0	4 (22.2)
Rash	1 (12.5)	0	3 (30.0)	0	4 (22.2)	0	4 (22.2)
Constipation	2 (25.0)	0	1 (10.0)	0	3 (16.7)	0	3 (16.7)
Diarrhea	1 (12.5)	0	2 (20.0)	0	3 (16.7)	0	3 (16.7)
Stomatitis	0	0	3 (30.0)	0	3 (16.7)	0	3 (16.7)
Cancer pain	1 (12.5)	0	1 (10.0)	1 (10.0)	2 (11.1)	1 (5.6)	3 (16.7)
Edema, peripheral	1 (12.5)	0	1 (10.0)	0	2 (11.1)	0	2 (11.1)
White blood cell count decreased	0	0	2 (20.0)	0	2 (11.1)	0	2 (11.1)
Neutrophil count decreased	0	0	1 (10.0)	1 (10.0)	1 (5.6)	1 (5.6)	2 (11.1)
Disseminated intravascular coagulation	0	0	0	1 (10.0)	0	1 (5.6)	1 (5.6)
Iron deficiency anemia	0	1 (12.5)	0	0	0	1 (5.6)	1 (5.6)
Hypothyroidism	0	0	1 (10.0)	0	1 (5.6)	0	1 (5.6)
Vomiting	1 (12.5)	0	0	0	1 (5.6)	0	1 (5.6)
General physical health deterioration	0	1 (12.5)	0	0	0	1 (5.6)	1 (5.6)
Cholangitis	0	0	0	1 (10.0)	0	1 (5.6)	1 (5.6)
Aspartate aminotransferase increased	0	0	0	1 (10.0)	0	1 (5.6)	1 (5.6)
Alanine aminotransferase increased	0	0	1 (10.0)	0	1 (5.6)	0	1 (5.6)
Hypoalbuminemia	0	0	1 (10.0)	0	1 (5.6)	0	1 (5.6)
Hypoglycemia	0	0	0	1 (10.0)	0	1 (5.6)	1 (5.6)
Peripheral sensory neuropathy	0	0	1 (10.0)	0	1 (5.6)	0	1 (5.6)
Headache	1 (12.5)	0	0	0	1 (5.6)	0	1 (5.6)
Hydronephrosis	0	1 (12.5)	0	0	0	1 (5.6)	1 (5.6)

TEAE, treatment-emergent adverse event.

The safety analysis set includes patients who received at least one dose of andecaliximab. TEAEs are AEs with onset dates on or after the administration of the first dose of the study drug (andecaliximab) and up to 30 days after the permanent withdrawal of andecaliximab or (if applicable) up to 5 months after the permanent withdrawal of nivolumab.

TEAEs occurred in all 8 and 10 patients in cohorts 1 and 4, respectively (table 3). In cohort 1 patients, grade ≥3 TEAEs included anemia (25%), iron deficiency anemia, fatigue, general physical health deterioration, decreased appetite, and hydronephrosis (each 12.5%). No DLTs were observed. In cohort 4 patients, grade ≥3 TEAEs included anemia (20%), disseminated intravascular coagulation, fatigue, cholangitis, neutropenia, increased aspartate aminotransferase, decreased appetite, hypoglycemia, and cancer pain (each 10%). Furthermore, in cohort 4 patients, the only andecaliximab-related AE of any grade was anemia (10%).

The TEAEs that were observed after andecaliximab monotherapy and andecaliximab in combination with nivolumab were well tolerated. No TEAE occurrence led to anticancer drug discontinuation or death.

### Efficacy

In cohort 1 patients, no responses to andecaliximab monotherapy were observed. In cohort 4, there were five PRs, with an ORR of 50% (5/10, 90% CI, 22% to 78%) in patients with measurable target lesions.

The median OS was 5.6 months (90% CI, 1.7 to 12.9) for cohort 1 and not reached in cohort 4. The median PFS was 1.4 months (90% CI, 0.5 to 5.4) and 4.6 months (90% CI, 0.9 to not reached) in cohort 1 and 4 patients, respectively.

### Biomarker assessments

**Figure 2:**
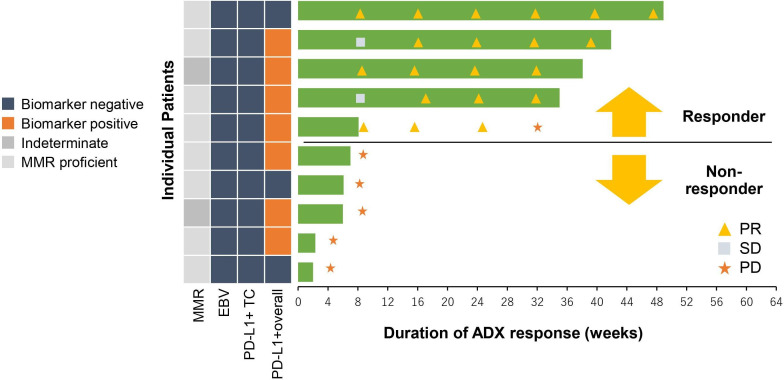
Cohort 4 biomarker status and best overall response. There were five responders: 80% (4/5) were mismatch repair-proficient, 100% (5/5) were Epstein–Barr virus-negative and programmed death ligand-1 (PD-L1) Tumor Proportion Score-negative, and 80% (4/5) were PD-L1 Combined Positive Score (CPS)-positive. However, the proportion of responders among PD-L1 CPS-positive patients was 4/7. ADX, andecaliximab; EBV, Epstein–Barr virus; MMP9, matrix metalloproteinase 9; MMR, mismatch repair; PD, pharmacodynamics; PD-L1, programmed death ligand-1; PR, partial response; SD, stable disease; TC, tumor cell.

For cohort 4 patients, MMR, PD-L1 staining, and EBV status were evaluated using baseline tumor tissues (table 4). No patients were unequivocally MMR-deficient (tested using MSH6 and PMS2), although two patients had indeterminate results. No patients were EBV-positive, and no patients had PD-L1 tumor cell staining ≥1% (Tumor Proportion Score (TPS)). Seven patients (70%) had PD-L1 overall cell staining ≥1% (Combined Positive Score (CPS)). TPS and CPS were defined as positive over 1% in their respective definitions (figure 2).

**Table 4:** Biomarker results in cohort 4

Patient	PD-L1 tumor status	PD-L1 overall status	MSH6	PMS2	MMR	EBV
1	Negative	Positive	Indeterminate	positive	Indeterminate	Negative
2	Negative	Negative	Positive	Positive	Proficient	Negative
3	Negative	Positive	Positive	Positive	Proficient	Negative
4	Negative	Negative	Positive	Positive	Proficient	Negative
5	Negative	Positive	Positive	Positive	Proficient	Negative
6	Negative	Negative	Positive	Positive	Proficient	Negative
7	Negative	Positive	Positive	Positive	Proficient	Negative
8	Negative	Positive	Positive	Positive	Proficient	Negative
9	Negative	Positive	Positive	Indeterminate	Indeterminate	Negative
10	Negative	Positive	Positive	Positive	Proficient	Negative

PD-L1 tumor status: ≥1% tumor cell staining. PD-L1 overall status:≥1% overall cell staining.

EBV, Epstein–Barr virus; MMR, mismatch repair; MSH6, human MutS homolog 6; PD-L1, programmed death ligand-1; PMS2, human PMS1 homolog 2.

Among the five responders in cohort 4, 80% (4/5) were MMR-proficient (MSS), 100% (5/5) were EBV-negative and PD-L1 TPS-negative, and 80% (4/5) were PD-L1 CPS-positive. From the PD-L1 CPS perspective, the ORRs of PD-L1 CPS-positive and PD-L1 CPS-negative patients were 57% (4/7) and 33% (1/3), respectively.

Biomarkers in patients with the longest durations of response were MSS, EBV negativity, and PD-L1 negativity (both CPS and TPS negativity).

Only three patients from cohort 4 had week 6 biopsies. All week 6 samples had sufficient tumor tissue to assess the biomarker status, but neither statistical analysis nor conclusions were available owing to the limited sample number.

## Discussion

Nivolumab is a fully human immunoglobulin (Ig) G4 monoclonal antibody inhibitor of PD-1^13^ and has clinical benefits against several cancers. The inhibition of the PD-1/PD-L1 axis causes effector T-cell reactivation within the tumor, leading to improved tumor cell killing. Decreasing the levels of immunosuppressive factors that limit the T-cell response in the tumor microenvironment or improving the trafficking of activated tumor-specific T cells to tumors may broaden and increase the antitumor response to PD-1/PD-L1 inhibition.^14^

Andecaliximab is a recombinant chimeric IgG4 monoclonal antibody that demonstrates a high affinity and selectivity for MMP9. A preclinical study reported that the treatment of established tumors with anti-MMP9 antibodies resulted in the upregulation of immune signature pathways in tumors and that the combination of anti-MMP9 and anti-PD-L1 antibodies increased T-cell receptor diversity and the proportion of memory/effector T cells in tumors.^15^ Based on these data, it was demonstrated that the inhibition of MMP9, a key component of the tumor-promoting and immune-suppressive myeloid inflammatory milieu, reduces tumor burden and promotes effector/memory T-cell infiltration and diversity when combined with an anti-PD-L1 antibody.^7 8^

Previous studies have shown an adequate clinical activity of andecaliximab in combination with modified oxaliplatin, leucovorin, and fluorouracil (mFOLFOX6) for advanced gastric cancer^12^ without additional toxicity; moreover, changes in serum biomarker levels suggested that these drugs may inhibit MMP9 enzymatic activity. Previous clinical studies using a pan-MMP inhibitor showed overexpression of MMP9 in patients with pancreatic cancer.^16 17^ A phase 1 study was conducted to examine the safety and efficacy of andecaliximab with gemcitabine plus nab-paclitaxel in patients with advanced pancreatic cancer.^18 19^ This combination therapy also resulted in favorable safety and clinical activity. These findings support that andecaliximab treatment inhibits MMP9 activity, which may lead to improved clinical outcomes.

In cohort 1 patients, no DLT was observed during the first 28 days of andecaliximab 800 mg Q2W. Based on the safety and PK profiles, we considered that an 800 mg dose of andecaliximab could be well tolerated, as previously reported.^12^ The PK after the administration of the first 800 mg dose of andecaliximab in Japanese patients enrolled in cohort 1 was consistent with that reported in previous studies on andecaliximab. Furthermore, the clearance and volume of the distribution of andecaliximab in Japanese patients following the administration of 800 mg andecaliximab were comparable with those previously reported in non-Japanese patients following the administration of 800 mg andecaliximab, indicating similar andecaliximab PK in Japanese and non-Japanese patients.^12^ In cohort 1 patients, circulating free MMP9 levels were below the limits of quantitation on-treatment at trough, therefore all MMP-9 was bound to andecaliximab for the duration of the Q2W dosing interval. The half-life of andecaliximab is 1 week; therefore, it can inhibit tumor growth as an anti-MMP9 agent.

AEs observed in the chemotherapy combination cohort were consistent with those previously reported with chemotherapy alone.^12^ We observed no serious AEs in patients who underwent andecaliximab monotherapy or andecaliximab–nivolumab combination therapy. Fifty per cent of patients experienced grade ≥3 TEAEs; however, these TEAEs were manageable. Therefore, these regimens were considered to be well tolerated without new or unexpected safety signals.

In terms of clinical response, the combination regimen was better than the monotherapy regimen. None of the patients treated with andecaliximab alone achieved a clinical response; however, five patients achieved PR with the combination regimen (ORR=50%). In the phase 3 ATTRACTION-2 trial, nivolumab resulted in a median PFS and OS of 1.61 months and 5.26 months, respectively, in patients with advanced gastric or GEJ cancer who had been previously treated with two or more chemotherapy regimens.^13^ In cohort 4 treated with an andecaliximab–nivolumab combination, the median PFS was 4.6 months, and the median OS was not reached. Therefore, the clinical activity of the andecaliximab–nivolumab combination regimen is promising. Considering these responses, a synergistic effect could be expected from an anti-MMP9–nivolumab combination.

In biomarker assessments of cohort 4, patients with MSS, PD-L1 negativity, and EBV negativity, which were previously reported to be biomarkers for less efficacy of nivolumab, showed responses when andecaliximab was combined with nivolumab. Appropriate biomarkers may be required to demonstrate the synergistic effect of an anti-MMP9 antibody and nivolumab.

The results of a phase 3 study on patients with gastric or GEJ adenocarcinoma (GS-US-296-1080) have been reported.^20^ Unfortunately, the first-line use of andecaliximab with mFOLFOX6 did not improve OS. However, the drug that was combined with andecaliximab and the treatment line were different from those in this study. This is the first report of a phase 1 study to evaluate the safety and clinical activity of andecaliximab combined with a PD-1 inhibitor in patients with gastric or GEJ adenocarcinoma.

Although this study included a small cohort, it demonstrated the efficacy of andecaliximab in combination with nivolumab in some patients. Further studies based on appropriate biomarkers are warranted.

## Conclusions

There were no clinically relevant differences in andecaliximab PK between Japanese patients with advanced or recurrent gastric or GEJ adenocarcinoma and non-Japanese patients with advanced solid tumors. Preliminary safety data demonstrated a manageable safety profile for andecaliximab alone and the andecaliximab–nivolumab combination. TEAEs in the andecaliximab–nivolumab combination treatment arm were consistent with those previously reported (GS-US-296-2013). The clinical activity of the andecaliximab–nivolumab regimen appears promising.

10.1136/jitc-2021-003518.supp2 Supplementary data

## Data Availability

Data are available in a public, open access repository.
