# Supplementary figures and images for: Safety and tolerability of andecaliximab as monotherapy and in combination with an anti-PD-1 antibody in Japanese patients with gastric or gastroesophageal junction adenocarcinoma: a phase 1b study
Source: J Immunother Cancer. 2022 Jan 6;10(1):e003518. doi: 10.1136/jitc-2021-003518 (PMC8739432; doi:10.1136/jitc-2021-003518)

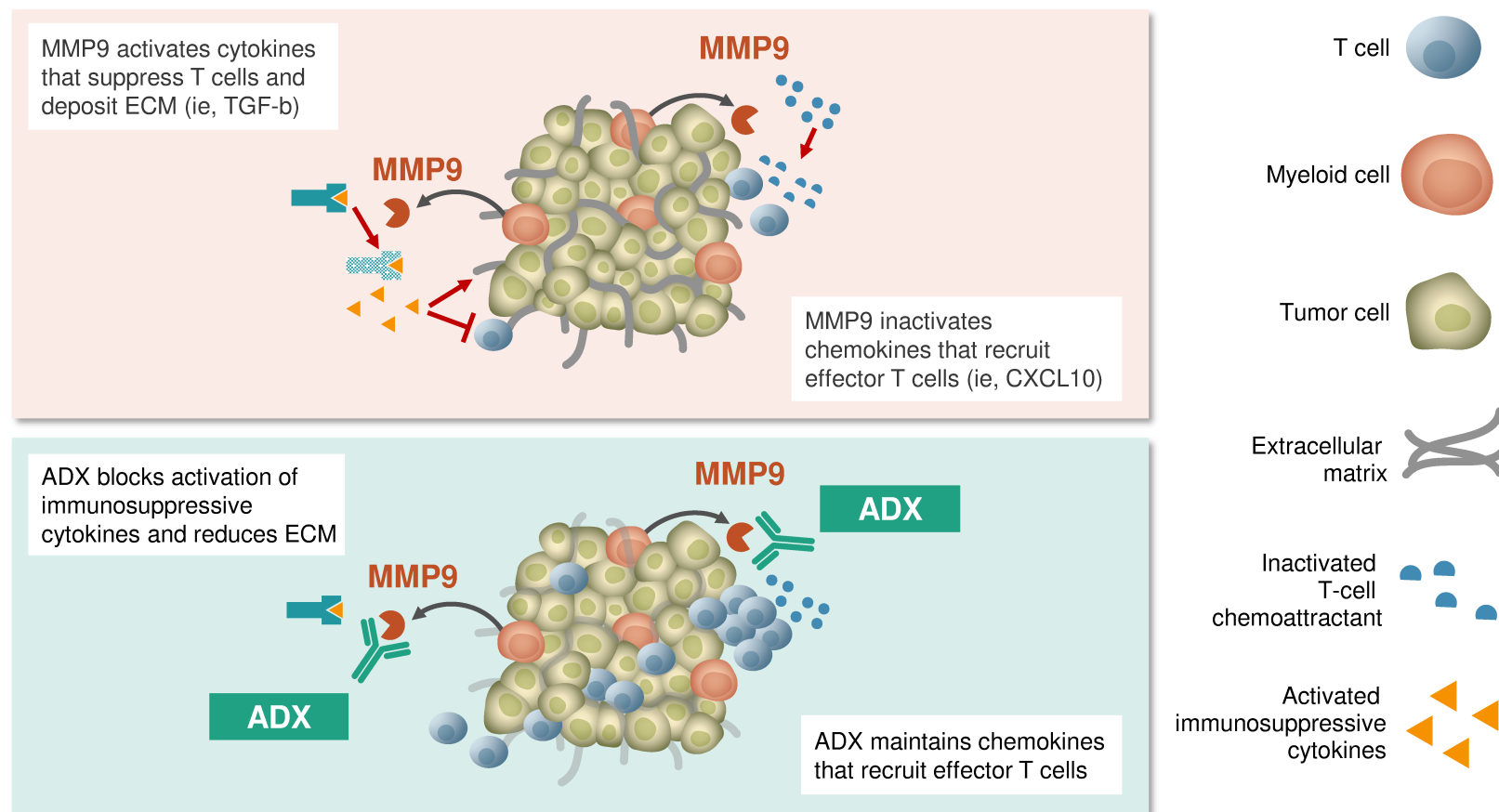

- CXCL10, C-X-C motif chemokine 10; ECM, extracellular matrix; TGF-  $\beta$ , transforming growth factor  $\beta$ .

Supplement: Supplementary data [file jitc-2021-003518supp003.pdf]
