# Supplementary material for: Safety and tolerability of andecaliximab as monotherapy and in combination with an anti-PD-1 antibody in Japanese patients with gastric or gastroesophageal junction adenocarcinoma: a phase 1b study
Source: J Immunother Cancer. 2022 Jan 6;10(1):e003518. doi: 10.1136/jitc-2021-003518 (PMC8739432; doi:10.1136/jitc-2021-003518)
Supplement: Supplementary data [file jitc-2021-003518supp001.pdf]

BMJ Publishing Group Limited (BMJ) disclaims all liability and responsibility arising from any reliance placed on this supplemental material which has been supplied by the author(s)


**Supplemental table 1** Prior anticancer therapy

| | Cohort 1 (n=8) | Cohort 4 (n=10) | Total (n=18) |
|---|---|---|---|
| Patients who received at least one prior anticancer therapy (%) | 8 (100) | 10 (100) | 18 (100) |
| Prior regimen | | | |
| Capecitabine / oxaliplatin | 0 | 3 (30.0) | 3 (16.7) |
| Capecitabine / cisplatin / trastuzumab | 0 | 1 (10.0) | 1 (5.6) |
| Cisplatin / S-1 / capecitabine / trastuzumab | 1 (12.5) | 0 | 1 (5.6) |
| Irinotecan | 2 (25.0) | 2 (20.0) | 4 (22.2) |
| Irinotecan / nimotuzumab | 1 (12.5) | 0 | 1 (5.6) |
| Oxaliplatin / levofolinate / fluorouracil | 1 (12.5) | 1 (10.0) | 2 (11.1) |
| Paclitaxel | 2 (25.0) | 0 | 2 (11.1) |
| Paclitaxel / ramucirumab | 5 (62.5) | 5 (50.0) | 10 (55.6) |
| S-1 | 3 (37.5) | 5 (50.0) | 8 (44.4) |
| S-1 / cisplatin | 4 (50.0) | 0 | 4 (22.2) |
| S-1 / cisplatin / dendritic cell vaccine | 1 (12.5) | 0 | 1 (5.6) |
| S-1 / oxaliplatin | 1 (12.5) | 3 (30.0) | 4 (22.2) |
| S-1 / cisplatin / docetaxel | 0 | 1 (10.0) | 1 (5.6) |
| S-1 / docetaxel / oxaliplatin | 0 | 1 (10.0) | 1 (5.6) |
| S-1 / cisplatin / ramucirumab | 1 (12.5) | 0 | 1 (5.6) |
| TAS118 / oxaliplatin | 0 | 1 (10.0) | 1 (5.6) |

S-1, tegafur / gimeracil / oteracil.

**Supplemental Table addition-1**: The probability of observing DLTs assuming the true DLT rate is more than 33%

| Number of patients with DLTs | Probability |
|---|---|
| >=2 | >=90% |
| >=3 | >=70% |
| >=4 | >=44% |



Yoshikawa AK, *et al. J Immunother Cancer* 2022; 10:e003518. doi: 10.1136/jitc-2021-003518

**Supplemental Table addition-2**: The confidence interval of observed ORR assuming the true ORR rate is 12%

| Observed objective response rate | 90% Confidence interval |
|---|---|
| 10% | (0.5%, 39.4%) |
| 20% | (3.7%, 50.7%) |
| 30% | (8.7%, 60.7%) |
| 40% | (15%, 69.6%) |
| 50% | (22.2%, 77.8%) |
| 60% | (30.4%, 85.0%) |
| 70% | (39.3%, 91.3%) |



Yoshikawa AK, *et al. J Immunother Cancer* 2022; 10:e003518. doi: 10.1136/jitc-2021-003518
