# Supplementary material for: Safety and tolerability of andecaliximab as monotherapy and in combination with an anti-PD-1 antibody in Japanese patients with gastric or gastroesophageal junction adenocarcinoma: a phase 1b study
Source: J Immunother Cancer. 2022 Jan 6;10(1):e003518. doi: 10.1136/jitc-2021-003518 (PMC8739432; doi:10.1136/jitc-2021-003518)
Supplement: Supplementary data [file jitc-2021-003518supp002.pdf]

BMJ Publishing Group Limited (BMJ) disclaims all liability and responsibility arising from any reliance placed on this supplemental material which has been supplied by the author(s)

PROTOCOL SYNOPSIS

Gilead Sciences, Inc.
333 Lakeside Drive
Foster City, CA 94404

**Study Title:**	A Phase 1b Study to Evaluate the Safety and Tolerability of Andecaliximab (GS-5745) as Monotherapy and in Combination with Anti-Cancer Agents in Japanese Subjects with Gastric or Gastroesophageal Junction Adenocarcinoma
**Clinical Trials.gov Identifier:**	02862535
**Study Centers Planned:**	Approximately 4 centers in Japan
**Objectives:**	The primary objective of this study is:<br>• To characterize the safety and tolerability of andecaliximab as monotherapy and in combination with anti-cancer agents in Japanese subjects with inoperable advanced or recurrent gastric or gastroesophageal junction (GEJ) adenocarcinoma<br>The secondary objectives of this study are:<br>• To characterize the pharmacokinetics (PK) of andecaliximab<br>• To evaluate the formation of anti-andecaliximab antibodies<br>The exploratory objectives of this study are:<br>• To evaluate andecaliximab pharmacodynamic biomarkers in the blood<br>• To explore biomarkers in tumor tissue<br>• To assess the therapeutic response of andecaliximab when used as monotherapy and in combination with anti-cancer agents
**Study Design:**	This is a Phase 1b, open-label, multicenter study to evaluate the safety and tolerability of andecaliximab as monotherapy and in combination with anti-cancer agents in Japanese subjects with inoperable advanced or recurrent gastric or GEJ adenocarcinoma.

The study will comprise 4 cohorts, 1 monotherapy cohort and 3 combination therapy cohorts.

- Cohort 1 is andecaliximab monotherapy
- Cohort 2 is combination therapy of andecaliximab with S-1 and cisplatin (SP)
- Cohort 3 is combination therapy of andecaliximab with S-1 and oxaliplatin (SOX)
- Cohort 4 is combination therapy of andecaliximab with nivolumab

Study Schema

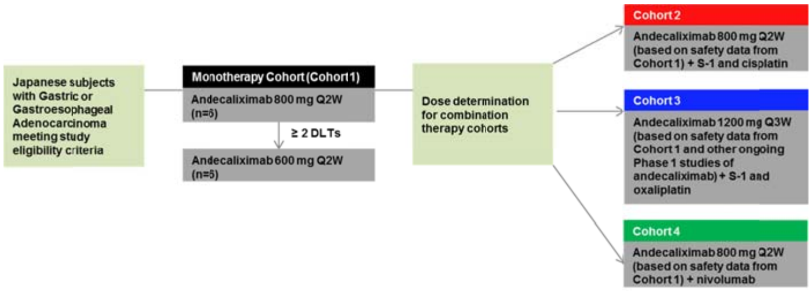

Abbreviations: DLT = dose limiting toxicity; Q2W = every 2 weeks; Q3W = every 3 weeks

Cohort 1: Andecaliximab Monotherapy

Up to 6 Japanese subjects with inoperable advanced or recurrent gastric or GEJ tumors will be enrolled to receive andecaliximab 800 mg via intravenous (IV) infusion over approximately 30 (± 5) minutes every two weeks (Q2W) until disease progression.

The dose limiting toxicity (DLT) assessment window is 28 days. The safety and tolerability of the 800 mg dose will be assessed after all 6 subjects have been followed for at least 28 days after the first infusion of andecaliximab. If 2 or more subjects within the cohort of 6 subjects experience DLTs during the first 28 days of andecaliximab dosing, up to 6 additional subjects will be enrolled at a reduced dose of 600 mg Q2W. If a subject is withdrawn from the study for any reason other than a DLT prior to completion of the DLT assessment window, a replacement subject will be enrolled. If 2 additional DLTs occur at 600 mg, andecaliximab will be considered unsafe and will be discontinued.

A DLT is a toxicity defined below, considered possibly related to andecaliximab, occurring during the DLT assessment window (Day 1 through Day 28).

- Grade 4 neutropenia (absolute neutrophil count [ANC] < 500/µL) for > 7 days, or febrile neutropenia (per the National Cancer Institute (NCI) Common Terminology Criteria for Adverse Events (CTCAE) Version 4.03 definition) regardless of duration.
- Primary prophylaxis with granulocyte-colony stimulating factor (G-CSF) is not permitted during the first cycle of study treatment. G-CSF may be used at any time at the discretion of the investigator in response to treatment emergent neutropenia. Subjects who develop Grade 4 neutropenia related to andecaliximab will be considered a DLT (subjects may receive G-CSF at the discretion of the investigator in response to treatment emergent neutropenia).
- Grade 4 thrombocytopenia, Grade 3 thrombocytopenia associated with bleeding, or Grade 3 or 4 thrombocytopenia requiring platelet transfusion
- Grade 3 or 4 non-hematologic toxicity (excluding rash, nausea, diarrhea, and vomiting if controlled with standard supportive care)
- Non-hematologic toxicity of ≥ Grade 2 (at any time during treatment) that, in the judgment of the investigator and the medical monitor, is dose-limiting
- Treatment delay of > 14 days due to unresolved toxicity

For certain toxicities such as laboratory assessments without a clear clinical correlate, a discussion between the investigator, medical monitor, and the sponsor may take place to determine if this adverse event (AE) should be assessed as a DLT necessitating dose reduction.

After all 6 subjects in Cohort 1 have completed the 28-day DLT assessment window and 0 or 1 out of 6 subjects experienced DLTs, an internal safety review team (SRT) will conduct a review of the safety and PK data from all subjects prior to proceeding with the combination therapy cohorts to evaluate andecaliximab with other anti-cancer agents.

The SRT completed their review of all available clinical safety and PK data for 6 DLT-evaluable subjects in Cohort 1 on 11 May 2017 and determined the dose for the combination therapy cohorts (Cohorts 2 and 4) to be 800 mg andecaliximab Q2W. See Section 1.3 for rationale of dose selection for Cohort 3 (1200 mg andecaliximab Q3W). The internal SRT consisted of Gilead study team members including the Medical Monitor, representatives from Clinical Operations, Drug Safety and Public Heath (DSPH), and Biostatistics.

Yoshikawa AK, *et al. J Immunother Cancer* 2022; 10:e003518. doi: 10.1136/jitc-2021-003518

Cohorts 2, 3, and 4: Andecaliximab Combination Therapy Cohorts

Japanese subjects with inoperable advanced or recurrent gastric or GEJ tumors will be enrolled in each combination therapy cohort to receive andecaliximab in combination with anti-cancer agents. Dosage and frequency of treatment in these cohorts will be as follows:

- Cohort 2: Combination therapy andecaliximab and SP
  - Andecaliximab 800 mg via IV infusion over approximately 30 (± 5) minutes Q2W until disease progression. The dose is based on safety data from Cohort 1. Andecaliximab treatment will be administered in 28-day cycles.
  - S-1 administered orally twice daily: dosage and regimen will be based on subject condition, investigator discretion, institutional practice and/or the in-country label
  - Cisplatin administered by IV infusion on Day 8 of every 5 weeks: dosage and regimen will be based on subject condition, investigator discretion, institutional practice and/or the in-country label
  - Up to 6 subjects will be enrolled in this cohort
- Cohort 3: Combination therapy andecaliximab and SOX
  - Andecaliximab 1200 mg via IV infusion over approximately 30 (± 5) minutes every 3 weeks (Q3W) until disease progression. The dose is based on safety data from Cohort 1 and other ongoing phase 1 studies of andecaliximab. Andecaliximab treatment will be administered in 21-day cycles.
  - S-1 administered orally twice daily at 80 mg/day for body surface area (BSA) $< 1.25m^2$, 100 mg/day for BSA ≥ 1.25 to $< 1.5m^2$, and 120 mg/day for BSA $\geq 1.5m^2$ for the first 14 days of the 21-day cycle
  - Oxaliplatin administered by IV infusion at 100 mg/m^2 over 2 hours on Day 1 of each 21-day cycle
  - Up to 10 subjects will be enrolled in this cohort
- Cohort 4: Combination therapy andecaliximab and nivolumab
  - Andecaliximab 800 mg via IV infusion over approximately 30 (± 5) minutes Q2W until disease progression. The dose is based on safety data from Cohort 1. Andecaliximab treatment will be administered in 28-day cycles.

- Nivolumab 3 mg/kg Q2W via IV infusion over 60 (± 5) minutes following the completion of andecaliximab administration. Dose is adjusted if the weight changes more than 10% from the baseline dosing weight.
- Up to 10 subjects will be enrolled in this cohort

For Cohorts 1, 2, and 4, computed tomography (CT) or magnetic resonance imaging (MRI) scans will be performed every 8 weeks to evaluate response to treatment by RECIST v1.1. For Cohort 4, response to treatment will also be evaluated using immune related response criteria recommendations {Wolchok 2009}.

For Cohort 3, CT or MRI scans will be performed every 9 weeks to evaluate response to treatment by RECIST v1.1.

The cohort dose levels for andecaliximab and associated combination anti-cancer agents are summarized below:

Cohort	Number of Subjects	Andecaliximab Dose	Andecaliximab Dosing Interval	Combination Anti-Cancer Agent(s)
1	6 to 12	800 mg or 600 mg	Q2W	N/A
2	Up to 6	800 mg	Q2W	SP
3	Up to 10	1200 mg	Q3W	SOX
4	Up to 10	800 mg	Q2W	Nivolumab

Abbreviations: SOX = S-1 and oxaliplatin; SP = S-1 and cisplatin; Q2W = every 2 weeks; Q3W = every 3 weeks

Number of Subjects Planned: Up to 38 subjects

Target Population: Japanese subjects ≥ 20 years of age with histologically confirmed inoperable advanced or recurrent gastric or GEJ adenocarcinoma

Duration of Treatment: For Cohorts 1, 2, and 4, each cycle will be 28 days and will continue in the absence of disease progression, unacceptable toxicity, withdrawal of consent, or other reasons specified in Section 3.5.

For Cohort 3, each cycle will be 21 days, to align with the SOX dosing schedule, and will continue in the absence of disease progression, unacceptable toxicity, withdrawal of consent, or other reasons specified in Section 3.5.

Diagnosis and Main Eligibility Criteria:	<u>Inclusion Criteria</u><br>Subjects must meet *all* of the following inclusion criteria to be eligible for participation in this study:<br>1) Male or female ≥ 20 years of age<br>2) Subjects must have been born in Japan and must not have lived outside of Japan for a period >1 year in the 5 years prior to Day 1<br>3) Subjects must be able to trace their maternal and paternal ancestry of parents and grandparents as ethnically Japanese<br>4) Histologically confirmed inoperable advanced gastric adenocarcinoma (including adenocarcinoma of the GEJ) or relapsed gastric adenocarcinoma<br>5) Cohorts 1 (andecaliximab monotherapy), 2 (combination therapy andecaliximab and SP) and 3 (combination therapy andecaliximab and SOX): Human Epidermal Growth Factor Receptor 2 (HER2)-negative tumor (primary tumor or metastatic lesion). Enrollment in Cohort 4 (combination therapy andecaliximab and nivolumab) is not restricted by HER2 status (subjects with HER2-positive, HER2-negative, or unknown HER2 status are eligible).<br>6) Cohort 1 (andecaliximab monotherapy): Prior antitumor therapy or cytotoxic chemotherapy is acceptable. Subjects who are not eligible to receive standard treatments should enroll on the study. All acute toxic effects of any prior antitumor therapy must be resolved to Grade ≤ 1 (or baseline) before the start of andecaliximab dosing (with the exception of alopecia [Grade 1 or 2 permitted] and neurotoxicity [Grade 1 or 2 permitted]).<br>7) Cohorts 2 (combination therapy andecaliximab and SP) and 3 (combination therapy andecaliximab and SOX): Prior antitumor therapy or cytotoxic chemotherapy for metastatic disease is **not** acceptable. Subjects must be chemo-naive in the metastatic setting. Subjects who relapse during adjuvant chemotherapy or within 180 days after adjuvant chemotherapy for their gastric cancer should not be enrolled.<br>8) Eastern Cooperative Oncology Group (ECOG) Performance Status of ≤ 1<br>9) Life expectancy of > 3 months in the opinion of the investigator

10) Adequate baseline organ function (within 28 days prior to Day 1) as shown in the following table:

Organ System	Parameter	Required Value
Hematopoietic	ANC	≥ 1.5 x 10^9/L
	Platelets	≥ 100 x 10^9/L
	Hemoglobin	Cohorts 1-3: ≥ 8.0 g/dL (not RBC transfusion dependent)
Hepatic	Serum total or conjugated bilirubin	≤ 1.5 x ULN
	Serum AST and ALT	≤ 2.5 x ULN (if liver metastases are present, ≤ 5 x ULN)
Renal	Serum Creatinine	Cohort 1: ≤ 1.5 x ULN<br>Cohorts 2 and 3: ≤ 1.0 x ULN

Abbreviations: ALT = alanine aminotransferase; ANC = absolute neutrophil count; AST = aspartate aminotransferase; RBC = red blood cell; ULN = upper limit of normal

11) Coagulation: International Normalized Ratio (INR) ≤ 1.5 (unless receiving anticoagulation therapy). Subjects on full-dose oral anticoagulation must be on a stable dose (minimum duration 14 days). If receiving warfarin, the subject must have an INR ≤ 3.0 and no active bleeding (ie, no bleeding within 14 days prior to first dose of study drug). Subjects on low molecular weight heparin will be allowed.

12) For female subjects of childbearing potential, willingness to use a protocol-recommended method of contraception from the screening visit throughout the study treatment period and defined periods following the last dose of andecaliximab and/or anti-cancer agent(s) (See Appendix 9)

13) For male subjects of childbearing potential having intercourse with females of childbearing potential, willingness to use a protocol-recommended method of contraception from the start of andecaliximab, throughout the study treatment period, and defined periods following the last dose of andecaliximab and/or anti-cancer agent(s), and to refrain from sperm donation from the start of andecaliximab, throughout the study treatment period, and defined periods following the last dose of andecaliximab and/or anti-cancer agent(s) (See Appendix 9)

14) Willingness to comply with scheduled visits, drug administration plan, imaging studies, laboratory tests, other study procedures, and study restrictions

15) Evidence of a personally signed informed consent form

16) In addition to the applicable criteria above, subjects in Cohort 4 (combination therapy andecaliximab and nivolumab) must meet *all* of the following inclusion criteria to be eligible for participation in this study:

a) Measureable gastric or GEJ adenocarcinoma according to RECIST v1.1

b) Subject must have progressed on at least 1 prior systemic therapy or line of treatment for unresectable/metastatic disease. All toxicities attributed to prior anti-cancer therapy other than alopecia or fatigue must have resolved to Grade ≤ 1 (NCI CTCAE Version 4) or baseline

c) Adequate baseline organ function (within 28 days prior to Day 1) as shown in the following table:

Organ System	Parameter	Required Value
Hematopoietic	ANC	≥ 1.5 x 10^9/L
	Platelets	≥ 100 x 10^9/L
	Hemoglobin	≥ 9.0 g/dL
Hepatic	Serum total or conjugated bilirubin	≤ 1.5 x ULN
	Serum AST and ALT	≤ 2.5 x ULN (if liver metastases are present, ≤ 5 x ULN)
Renal	Creatinine Clearance	Creatinine clearance (CLcr) ≥ 60 mL/min, estimated based on the Cockroft-Gault formula or measured based on 24 hour urine collection or other reliable method

Abbreviations: ALT = alanine aminotransferase; ANC = absolute neutrophil count; AST = aspartate aminotransferase; CLcr = creatinine clearance; RBC = red blood cell; ULN = upper limit of normal

d) Subjects not receiving anticoagulant medication must have an activated partial thromboplastin (aPTT) ≤ 1.5 x ULN. The use of full-dose oral or parenteral anticoagulants is permitted as long as the aPTT is within therapeutic limits (according to the medical standard in the institution) and the subject has been on stable dose of anticoagulants for at least 1 week at the time of enrollment

e) Thyroid function tests (thyroid-stimulating hormone (TSH), T3, free T4) should be within normal limits. Subjects with underlying thyroid disease are eligible if they are receiving appropriate medication and are clinically stable.

Exclusion Criteria

Subjects who meet *any* of the following exclusion criteria will not be enrolled in this study:

1) History or evidence of a clinically significant disorder, condition, or disease that, in the opinion of the investigator and medical monitor would pose a risk to subject safety or interfere with the study evaluations, procedures, or completion
2) Pregnant or lactating. Enrollment of lactating females after discontinuation of breastfeeding is not acceptable.
3) Subjects with known central nervous system (CNS) metastases, unless metastases are treated and stable and the subject does not require systemic steroids
4) Radiotherapy within 28 days of Day 1; subjects given palliative radiotherapy to peripheral sites (eg, bone metastasis) may enter the study before 28 days have elapsed if subject has recovered from any acute reversible effects
5) Myocardial infarction, symptomatic congestive heart failure (New York Heart Association Classification > Class II), unstable angina, or serious uncontrolled cardiac arrhythmia within the last 6 months of Day 1
6) History of major surgery within 28 days of Day 1
7) Serious systemic fungal, bacterial, viral, or other infection that is not controlled or requires IV antibiotics
8) Cohort 1 (andecaliximab monotherapy): Anti-tumor therapy (chemotherapy, antibody therapy, molecular targeted therapy) within 28 days or 5 half-lives, whichever is shorter, of Day 1 (6 weeks for nitrosoureas, mitomycin C, or molecular agents with $t_{½}$ > 10 days)
9) Clinically significant bleeding within 28 days of Day 1
10) Subjects known to be positive for human immunodeficiency virus (HIV), hepatitis C infection (per local standard diagnostic criteria), or acute or chronic hepatitis B infection (per local standard diagnostic criteria)
11) Known hypersensitivity to any of the study drugs or components or to Chinese hamster ovary cell products or to recombinant human or humanized antibodies

12) History of a concurrent or second malignancy except for adequately treated local basal cell or squamous cell carcinoma of the skin; cervical carcinoma in situ; superficial bladder cancer; asymptomatic prostate cancer without known metastatic disease, with no requirement for therapy or requiring only hormonal therapy, and with normal prostate-specific antigen for ≥ 1 year prior to Day 1; adequately treated Stage 1 or 2 cancer currently in complete remission; or any other cancer that has been in complete remission for ≥ 5 years

13) Known alcohol or drug abuse or any other medical or psychiatric condition which contraindicates participation in the study

14) Subject is expected to require any form of systemic or localized antineoplastic therapy while on study

15) In addition to the applicable criteria above, subjects in Cohort 4 (combination therapy andecaliximab and nivolumab) who meet *any* of the following exclusion criteria will not be enrolled in this study:

   a) Subjects who have received only neoadjuvant or adjuvant therapy for gastric adenocarcinoma

   b) Chronic daily treatment with oral corticosteroids (dose of > 10 mg/day prednisone equivalent) or other immunosuppressive medications within 14 days of Day 1. Inhaled steroids and short courses of oral steroids for anti-emesis or as an appetite stimulant are allowed.

   c) Anti-tumor therapy (chemotherapy, antibody therapy, molecular targeted therapy) within 28 days or 5 half-lives, whichever is shorter, of Day 1 (6 weeks for nitrosoureas, mitomycin C, or molecular agents with $t_{½}$ > 10 days)

   d) Prior treatment with anti-CTLA-4 agents (eg, ipilimumab), anti-PD-1 or anti-PD-L1 agents (eg, pembrolizumab, nivolumab, atezolizumab), anti-PD-L2 agents, anti-MMP agents, or other immunomodulatory therapies

   e) Prior therapy with anti-tumor vaccines or other immuno-modulatory antitumor agents

   f) Current or history of pneumonitis or interstitial lung disease

   g) Active known or suspected autoimmune disease. Subjects with vitiligo, type I diabetes mellitus, residual hypothyroidism requiring hormone replacement, or conditions not expected to recur in the absence of an external trigger are permitted to enroll

   h) History of bone marrow, stem cell, or allogeneic organ transplantation

Study Procedures: Screening:

Screening will commence with obtaining the subject's signed informed consent, and will occur up to 28 days prior to the first dosing of andecaliximab on Day 1. Screening procedures will include the following: medical history review, physical exam (PE), vital signs, 12-lead electrocardiogram (ECG), ECOG Performance Status, prior/concomitant medication review, blood collection for pregnancy test (females), urinalysis, chemistry, hematology, coagulation, thyroid function tests (for Cohort 4 only: combination therapy andecaliximab and nivolumab), biomarkers, AE assessment, pre-treatment fresh biopsy collection, and CT or MRI (scans obtained as part of standard medical practice up to 28 days prior to Day 1 are acceptable). Baseline tumor lesions will be measured and characterized prior to Day 1 to assess subject disease status prior to beginning treatment.

Treatment: Cohort 1: Andecaliximab Monotherapy

Treatment will occur over cycles comprised of 28 days. Subjects who meet eligibility will undergo CT or MRI scans every 8 weeks. Beginning with Day 1 of Cycle 1, subjects will receive andecaliximab 800 mg by IV infusion over approximately 30 minutes Q2W for a total of 2 infusions per cycle (Day 1 and Day 15 of each 28-day cycle). Safety and efficacy assessments will occur on an outpatient basis including assessment of tumor response, physical exam, vitals, ECG, collection of blood samples, urine pregnancy, urinalysis, and assessment of AEs at the protocol specified time points.

The DLT assessment window is 28 days. The safety and tolerability of the 800 mg dose will be assessed after all 6 subjects have been followed for at least 28 days after the first infusion of andecaliximab. If 2 or more subjects within the cohort of 6 subjects experience DLTs during the first 28 days of andecaliximab dosing, 6 additional subjects will be enrolled at a reduced dose of 600 mg Q2W. If 2 additional DLTs occur at 600 mg, andecaliximab will be considered unsafe and will be discontinued. Therefore, a total of up to 12 subjects may be enrolled in Cohort 1.

Once all subjects in Cohort 1 have completed the 28-day DLT assessment window and 1 or 0 out of 6 subjects experienced DLTs, an internal SRT will conduct a review of the safety and PK data from all subjects prior to proceeding with the combination therapy cohorts to evaluate andecaliximab with other anti-cancer agents.

The SRT completed their review of all available clinical safety and PK data for 6 DLT-evaluable subjects in Cohort 1 on 11 May 2017 and determined the dose for the combination therapy cohorts (Cohorts 2 and 4) to be 800 mg andecaliximab Q2W. See Section 1.3 for rationale of

dose selection for Cohort 3 (1200 mg andecaliximab Q3W). The internal SRT consisted of Gilead study team members including the Medical Monitor, representatives from Clinical Operations, Drug Safety and Public Heath (DSPH), and Biostatistics.

Treatment: Cohorts 2, 3, and 4: Andecaliximab Combination Therapy Cohorts

- Cohort 2: combination therapy andecaliximab and SP:
  - Treatment will be administered in 28-day cycles. Subjects who meet eligibility will undergo CT or MRI scans every 8 weeks. Beginning with Day 1 of Cycle 1, subjects will receive andecaliximab 800 mg by IV infusion over approximately 30 (± 5) minutes Q2W, for a total of 2 infusions per cycle (Day 1 and Day 15 of each 28-day cycle).
  - Subjects will also receive S-1 orally twice daily and cisplatin administered by IV infusion on Day 8 of every 5 weeks. The dosage and regimen of chemotherapy will be based on subject condition, investigator discretion, institutional practice and/or the in-country label (see below for the proposed regimen).

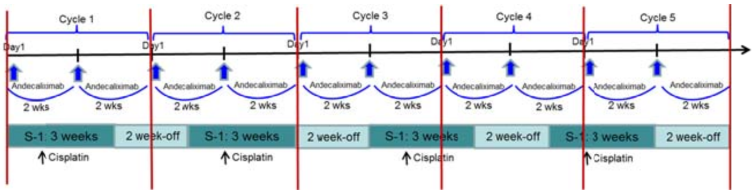

- Cohort 3: combination therapy andecaliximab and SOX:
  - Treatment will be administered in 21-day cycles. Subjects who meet eligibility will undergo CT or MRI scans every 9 weeks. Beginning with Day 1 of Cycle 1, subjects will receive andecaliximab 1200 mg via IV infusion over approximately 30 (± 5) minutes Q3W, for a total of 1 infusion per cycle (Day 1 of each 21-day cycle).
  - S-1 administered orally twice daily at 80 mg/day for BSA $< 1.25m^2$, 100 mg/day for BSA ≥ 1.25 to $< 1.5m^2$, and 120 mg/day for BSA $\geq 1.5m^2$ for the first 14 days of the 21-day cycle (see below for regimen)
  - Oxaliplatin administered by IV infusion at 100 mg/m^2 over 2 hours on Day 1 of each 21-day cycle (see below for regimen)

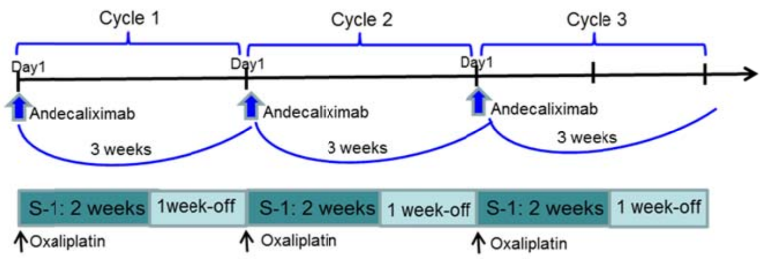

- Cohort 4: combination therapy andecaliximab and nivolumab:
  - Treatment will be administered in 28-day cycles. Subjects who meet eligibility will undergo CT or MRI scans every 8 weeks. Beginning with Day 1 of Cycle 1, subjects will receive andecaliximab 800 mg via IV infusion over approximately 30 (± 5) minutes Q2W, for a total of 2 infusions per cycle (Day 1 and Day 15 of each 28-day cycle) (see below for regimen).
  - Nivolumab 3 mg/kg Q2W via IV infusion over 60 (± 5) minutes following the completion of andecaliximab administration for a total of 2 infusions per cycle (Day 1 and Day 15 of each 28-day cycle, see below for regimen). Dose is adjusted if the weight changes more than 10% from the baseline dosing weight.

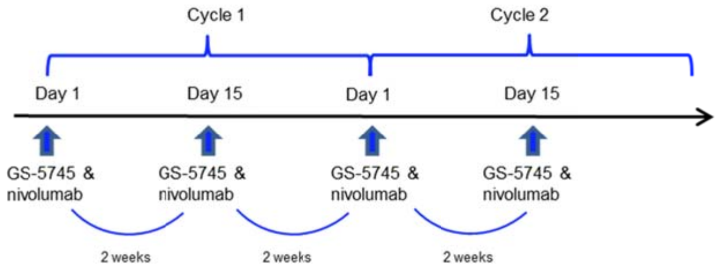

Safety and efficacy assessments will occur on an outpatient basis including assessment of tumor response, physical exam, vitals, ECG, collection of blood samples, urine pregnancy, urinalysis, and assessment of AEs at the protocol specified time points.

Continuation of Treatment:

A subject who does not show evidence of disease progression by clinical assessment or by CT or MRI may continue receiving treatment until disease progression (clinical or radiographic), unacceptable toxicity, withdrawal of consent, or other reasons specified in Section 3.5. If corresponding anti-cancer agent treatment is permanently discontinued (SP, SOX, or nivolumab), andecaliximab must be discontinued. If andecaliximab is discontinued for reasons other than disease progression, subjects may continue to receive corresponding anti-cancer agent treatment (SP, SOX, or nivolumab) until disease progression.

	CT or MRI for assessment of tumor status will be conducted every 8 weeks for Cohorts 1, 2, and 4, and every 9 weeks for Cohort 3. However, tumor response may be assessed prior to the specified every 8- or 9-week time point if clinically indicated.
**Test Product, Dose, and Mode of Administration:**	Andecaliximab is formulated as a sterile, aqueous buffered solution and is stored at 2 to 8° C in single-use 10 ml vials containing 400 mg andecaliximab at a concentration of 40 mg/ml.<br>Subjects will be administered 800 mg or 600 mg andecaliximab via IV infusion over approximately 30 minutes Q2W or 1200 mg andecaliximab via IV infusion over approximately 30 minutes Q3W.
**Criteria for Evaluation:**	All subjects who meet eligibility criteria, have signed a consent form, and have begun treatment, will be evaluated for response.
Safety	Safety will be evaluated by assessment of clinical laboratory tests, physical examination, 12-lead ECG, vital sign measurements, and by the incidence of AEs.
Pharmacokinetics/ Pharmacodynamics	Plasma drug concentrations and exploratory pharmacodynamic biomarkers will be analyzed.
Efficacy	The exploratory efficacy endpoints will include:<br>• Objective response rate (ORR) – defined as the proportion of subjects who achieve a Complete Response (CR) or Partial Response (PR) as assessed by RECIST v1.1<br>• Progression free survival (PFS) – defined as the interval from the first dose of andecaliximab to the earlier of the first documentation of definitive disease progression or death from any cause<br>• Overall survival (OS) – defined as the time from date of first dose of andecaliximab to death from any cause<br>• Duration of response (DOR) – defined as the time from the first documentation of CR or PR to the earlier of the first documentation of definitive disease progression or death from any cause for the responders<br>• Disease control rate (DCR) – defined as the proportion of subjects who achieve a CR, PR, Stable Disease (SD), or Non-CR/Non-PD (NN) as assessed by RECIST v1.1<br>• Time to response (TTR) – defined as the time from first dose of andecaliximab to the first documentation of CR or PR for the responders<br>• Change in tumor size – defined as the percent change from baseline in the sum of the diameters of target lesions

Statistical Methods:

Appropriate data analysis sets will be defined.

The Safety Analysis Set is defined as all subjects who receive at least 1 infusion at any dose of andecaliximab.

The Pharmacokinetic/Pharmacodynamic (PK/PD) Analysis Set is defined as all subjects in the Safety Analysis Set who have the necessary baseline and on-study measurements to provide interpretable results for specific parameters of interest.

Subject characteristics and study results will be described and summarized by treatment dose level/cohort for the relevant analysis sets. Descriptive statistics including sample size, mean, median, standard deviation, and ranges will be summarized for continuous variables, and categorical variables will be summarized using frequency counts and percentages.

The Safety Analysis Set will be used for both the safety and efficacy analyses. Andecaliximab plasma concentrations and parameters will be described and summarized using the PK Analysis Set.

Sample size

The sample size of 6 subjects in Cohort 1 (andecaliximab monotherapy) allows a relatively high probability (> 65%) to observe 2 or more subjects with DLT when the true underlying probability of DLT is greater than 33.3% at current dose level.

Up to 6 subjects will be enrolled in Cohort 1 to receive 800 mg andecaliximab Q2W, and based on safety assessments, an additional 6 subjects may be enrolled in Cohort 1 to receive 600mg andecaliximab Q2W prior to proceeding with the combination therapy cohorts. Based on the dose level selected by Cohort 1, up to 6 subjects will be enrolled in Cohort 2 (combination therapy andecaliximab and SP), and up to 10 subjects will be enrolled in Cohorts 3 (combination therapy andecaliximab and SOX) and 4 (combination therapy andecaliximab and nivolumab), respectively.

Therefore, up to 38 subjects will be enrolled in the study.

This study will be conducted in accordance with the guidelines of Good Clinical Practice (GCP) including archiving of essential documents.

BMJ Publishing Group Limited (BMJ) disclaims all liability and responsibility arising from any reliance placed on this supplemental material which has been supplied by the author(s)

BMJ Publishing Group Limited (BMJ) disclaims all liability and responsibility arising from any reliance placed on this supplemental material which has been supplied by the author(s)

BMJ Publishing Group Limited (BMJ) disclaims all liability and responsibility arising from any reliance placed on this supplemental material which has been supplied by the author(s)

BMJ Publishing Group Limited (BMJ) disclaims all liability and responsibility arising from any reliance placed on this supplemental material which has been supplied by the author(s)

BMJ Publishing Group Limited (BMJ) disclaims all liability and responsibility arising from any reliance placed on this supplemental material which has been supplied by the author(s)

BMJ Publishing Group Limited (BMJ) disclaims all liability and responsibility arising from any reliance placed on this supplemental material which has been supplied by the author(s)

Appendix 1. Investigator Signature Page

Supplemental material
BMJ Publishing Group Limited (BMJ) disclaims all liability and responsibility arising from any reliance placed on this supplemental material which has been supplied by the author(s)
J Immunother Cancer

GILEAD SCIENCES, INC.
333 LAKESIDE DRIVE
FOSTER CITY CA 94404

STUDY ACKNOWLEDGEMENT

A Phase 1b Study to Evaluate the Safety and Tolerability of Andecaliximab (GS-5745) as Monotherapy and in Combination with Chemotherapy in Japanese Subjects with Gastric or Gastroesophageal Junction Adenocarcinoma

GS-US-296-1884, Amendment 3, 14 July 2017

This protocol has been approved by Gilead Sciences, Inc. The following signature documents this approval.

VOSGANIAN, G.
Greg Vosganian, MD
Medical Monitor

Signature

14JUL2017
Date

INVESTIGATOR STATEMENT

I have read the protocol, including all appendices, and I agree that it contains all necessary details for me and my staff to conduct this study as described. I will conduct this study as outlined herein and will make a reasonable effort to complete the study within the time designated.

I will provide all study personnel under my supervision copies of the protocol and access to all information provided by Gilead Sciences, Inc. I will discuss this material with them to ensure that they are fully informed about the drugs and the study.

Principal Investigator Name (Printed)

Signature

Yoshikawa AK, *et al. J Immunother Cancer* 2022; 10:e003518. doi: 10.1136/jitc-2021-003518

Date

Site Number

BMJ Publishing Group Limited (BMJ) disclaims all liability and responsibility arising from any reliance placed on this supplemental material which has been supplied by the author(s)

BMJ Publishing Group Limited (BMJ) disclaims all liability and responsibility arising from any reliance placed on this supplemental material which has been supplied by the author(s)

BMJ Publishing Group Limited (BMJ) disclaims all liability and responsibility arising from any reliance placed on this supplemental material which has been supplied by the author(s)

BMJ Publishing Group Limited (BMJ) disclaims all liability and responsibility arising from any reliance placed on this supplemental material which has been supplied by the author(s)

BMJ Publishing Group Limited (BMJ) disclaims all liability and responsibility arising from any reliance placed on this supplemental material which has been supplied by the author(s)

BMJ Publishing Group Limited (BMJ) disclaims all liability and responsibility arising from any reliance placed on this supplemental material which has been supplied by the author(s)

BMJ Publishing Group Limited (BMJ) disclaims all liability and responsibility arising from any reliance placed on this supplemental material which has been supplied by the author(s)

BMJ Publishing Group Limited (BMJ) disclaims all liability and responsibility arising from any reliance placed on this supplemental material which has been supplied by the author(s)

BMJ Publishing Group Limited (BMJ) disclaims all liability and responsibility arising from any reliance placed on this supplemental material which has been supplied by the author(s)

BMJ Publishing Group Limited (BMJ) disclaims all liability and responsibility arising from any reliance placed on this supplemental material which has been supplied by the author(s)

BMJ Publishing Group Limited (BMJ) disclaims all liability and responsibility arising from any reliance placed on this supplemental material which has been supplied by the author(s)
